# An ultrapotent pan-β-coronavirus lineage B (β-CoV-B) neutralizing antibody locks the receptor-binding domain in closed conformation by targeting its conserved epitope

**DOI:** 10.1007/s13238-021-00871-6

**Published:** 2021-09-23

**Authors:** Zezhong Liu, Wei Xu, Zhenguo Chen, Wangjun Fu, Wuqiang Zhan, Yidan Gao, Jie Zhou, Yunjiao Zhou, Jianbo Wu, Qian Wang, Xiang Zhang, Aihua Hao, Wei Wu, Qianqian Zhang, Yaming Li, Kaiyue Fan, Ruihong Chen, Qiaochu Jiang, Christian T. Mayer, Till Schoofs, Youhua Xie, Shibo Jiang, Yumei Wen, Zhenghong Yuan, Kang Wang, Lu Lu, Lei Sun, Qiao Wang

**Affiliations:** 1 Key Laboratory of Medical Molecular Virology (MOE/NHC/CAMS), School of Basic Medical Sciences; Shanghai Institute of Infectious Disease and Biosecurity; the Fifth People’s Hospital of Shanghai; Shanghai Key Laboratory of Medical Epigenetics, International Co-laboratory of Medical Epigenetics and Metabolism (Ministry of Science and Technology); Institutes of Biomedical Sciences; Biosafety Level 3 Laboratory, Shanghai Medical College, Fudan University, Shanghai, 200032 China; 2 CAS Key Laboratory of Infection and Immunity, National Laboratory of Macromolecules, Institute of Biophysics, Chinese Academy of Sciences, Beijing, 100101 China; 3 Experimental Immunology Branch, Center for Cancer Research, National Cancer Institute, National Institutes of Health, Bethesda, MD 20892 USA; 4 GSK Vaccines, 1300 Wavre, Belgium

**Keywords:** SARS-CoV-2, neutralizing antibody, receptor-binding domain, XG014, antibody-dependent cell-cell fusion

## Abstract

New threats posed by the emerging circulating variants of SARS-CoV-2 highlight the need to find conserved neutralizing epitopes for therapeutic antibodies and efficient vaccine design. Here, we identified a receptor-binding domain (RBD)-binding antibody, XG014, which potently neutralizes β-coronavirus lineage B (β-CoV-B), including SARS-CoV-2, its circulating variants, SARS-CoV and bat SARSr-CoV WIV1. Interestingly, antibody family members competing with XG014 binding show reduced levels of cross-reactivity and induce antibody-dependent SARS-CoV-2 spike (S) protein-mediated cell-cell fusion, suggesting a unique mode of recognition by XG014. Structural analyses reveal that XG014 recognizes a conserved epitope outside the ACE2 binding site and completely locks RBD in the non-functional “down” conformation, while its family member XG005 directly competes with ACE2 binding and position the RBD “up”. Single administration of XG014 is effective in protection against and therapy of SARS-CoV-2 infection *in vivo*. Our findings suggest the potential to develop XG014 as pan-β-CoV-B therapeutics and the importance of the XG014 conserved antigenic epitope for designing broadly protective vaccines against β-CoV-B and newly emerging SARS-CoV-2 variants of concern.

**Supplementary Information:**

The online version contains supplementary material available at 10.1007/s13238-021-00871-6.

## Introduction

The pandemic of coronavirus disease of 2019 (COVID-19) is caused by the severe acute respiratory syndrome coronavirus 2 (SARS-CoV-2) with more than 190 million confirmed infections and a death toll exceeding 4 million (https://www.who.int/). Since the outbreak of COVID-19, great efforts have been made globally to develop effective countermeasures against SARS-CoV-2 infection, such as vaccines and therapeutic antibodies (Krammer, 2020; Lurie et al., 2020; Baden et al., 2021; Weinreich et al., 2021; Chen et al., 2021a). To date, several vaccines and monoclonal antibodies have been tested in clinical trials or have been granted authorization for emergency use in many countries. Several types of vaccines, including mRNA-, adenoviral vector-based and inactivated virus vaccines, induce strong immune responses (Corbett et al., 2020; Gao et al., 2020; Krammer, 2020; Mercado et al., 2020; Poland et al., 2020), while several human neutralizing monoclonal antibodies isolated from convalescent patients, such as REGN10987, REGN10933, LY-CoV555, C144 and C135, effectively neutralize SARS-CoV-2 for prophylaxis and treatment (Alsoussi et al., 2020; Baum et al., 2020; Robbiani et al., 2020; Rogers et al., 2020; Shi et al., 2020; Zost et al., 2020; Wang et al., 2020a; Andreano et al., 2021; Group et al., 2021; Weinreich et al., 2021; Chen et al., 2021a).

Over the past few months, the emergence and circulation of several SARS-CoV-2 lineage B variants, such as B.1.1.7 (Alpha, WHO label), B.1.351 (Beta, WHO label), P.1 (Gamma, WHO label) and B.1.617.2 (Delta, WHO label) identified in the United Kingdom, South Africa, Brazil and India, respectively, have posed a new challenge (Grubaugh et al., 2021; Harvey et al., 2021; Lopez Bernal et al., 2021; Planas et al., 2021; Supasa et al., 2021; Xie et al., 2021; Wang et al., 2021b). Current studies have shown that the mutations in these SARS-CoV-2 variants could increase the affinity of the receptor-binding domain (RBD) to its cellular receptor angiotensin-converting enzyme-2 (ACE2) and confer resistance to vaccine sera and many monoclonal antibodies (Hou et al., 2020; Korber et al., 2020; Starr et al., 2020; Yurkovetskiy et al., 2020; Greaney et al., 2021; Gupta, 2021; Liu et al., 2021; Plante et al., 2021; Wang et al., 2021c, d). Convalescent plasma or sera from individuals vaccinated by mRNA vaccines or inactivated-virus vaccines show a significant reduction in neutralizing activity against these emerging circulating variants (Weisblum et al., 2020; Wibmer et al., 2021; Chen et al., 2021b; Wang et al., 2021a, 2021d). Such immune escapes have also been reported for several monoclonal antibodies that are in the clinic such as LY-CoV555, CB6 and REGN10933 (Wang et al., 2021b, d). These variants indicate continuous antigenic drift of SARS-CoV-2 and highlight its adaptability to the human host (Wang et al., 2021b). Therefore, the identification of highly conserved neutralizing epitopes could help generate more broadly protective vaccines and therapeutic drugs, but little is known about the identity and biology of such cross-neutralizing epitopes.

Here, we evaluated the neutralizing activity of four monoclonal antibodies targeting four non-overlapping RBD epitopes that we isolated from a convalescent individual previously infected with SARS-CoV-2 (Zhou et al., 2021b). We found that one of these antibodies, XG014, is ultrapotent and broadly neutralizing, without enhancing S protein-mediated membrane fusion. Cryo-electron microscopy (Cryo-EM) showed that XG014 recognizes a conserved epitope outside of the receptor-binding motif (RBM) in RBD, and that it locks all three RBDs of the S trimer in the “down” or “closed” conformation, sterically hindering receptor binding. Epitope comparison with another antibody, XG005, which belongs to the same epitope group with XG014 but enhances S protein-mediated membrane fusion, provides a structural explanation for the potent and broad cross-neutralizing activity of XG014. Finally, XG014 was effective for SARS-CoV-2 prophylaxis and treatment in human ACE2-transgenic (hACE2-Tg) mice, suggesting the potential for XG014 to be developed as a broadly effective therapeutic agent.

## Results

### Four groups of neutralizing epitopes on SARS-CoV-2 RBD

**Figure 1:**
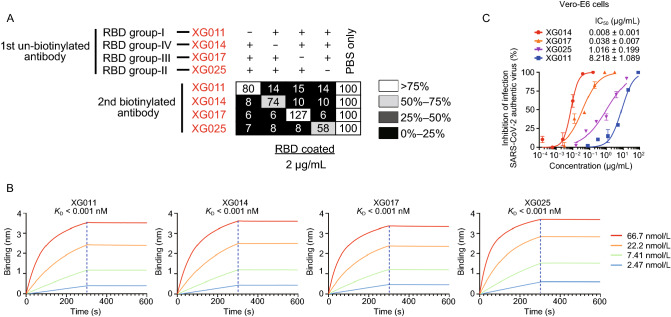
**Four monoclonal antibodies with non-overlapping epitopes**. (A) Competition ELISA for the four monoclonal antibodies against recombinant SARS-CoV-2 RBD protein. The 1st antibodies were unbiotinylated and added in combinations of three antibodies in each well to block the coated RBD, while respective 2nd biotinylated antibodies were used each on its own to detect binding. Results of competition ELISA shown as percent of binding by the 2nd biotinylated antibodies and illustrated in the following colors: black, 0%–25%; dark gray, 26%–50%; light gray, 51%–75%; white, >76%. Representative of two experiments. (B) BLI sensorgrams and kinetics of monoclonal antibodies binding to biotinylated RBD protein of SARS-CoV-2. Four fitting curves are shown with the *K*_D_ values. (C) *In vitro* neutralization assay using authentic SARS-CoV-2 virus in the presence of different concentrations of antibodies XG011, XG014, XG017, XG025. The infection rate was determined by plaque reduction assay. Duplicates of neutralization are presented as means ± SEM

We have previously identified monoclonal antibodies recognizing four groups of non-overlapping RBD epitopes (Zhou et al., 2021b). Four representative neutralizing antibodies (XG011, XG014, XG017, XG025) from each epitope group (Fig. 1A) were chosen for further study owing to their prominent neutralizing activity against SARS-CoV-2. All four monoclonal antibodies exhibited high binding affinity to the RBD of S protein with K_D_ values lower than 0.001 nM (Fig. 1B), and they effectively neutralized authentic SARS-CoV-2 virus with 50% inhibition concentration (IC_50_) values of 8.218 µg/mL, 0.008 µg/mL, 0.038 µg/mL, and 1.016 µg/mL, respectively (Fig. 1C).

### Potent and broad neutralizing activity of XG014

**Figure 2:**
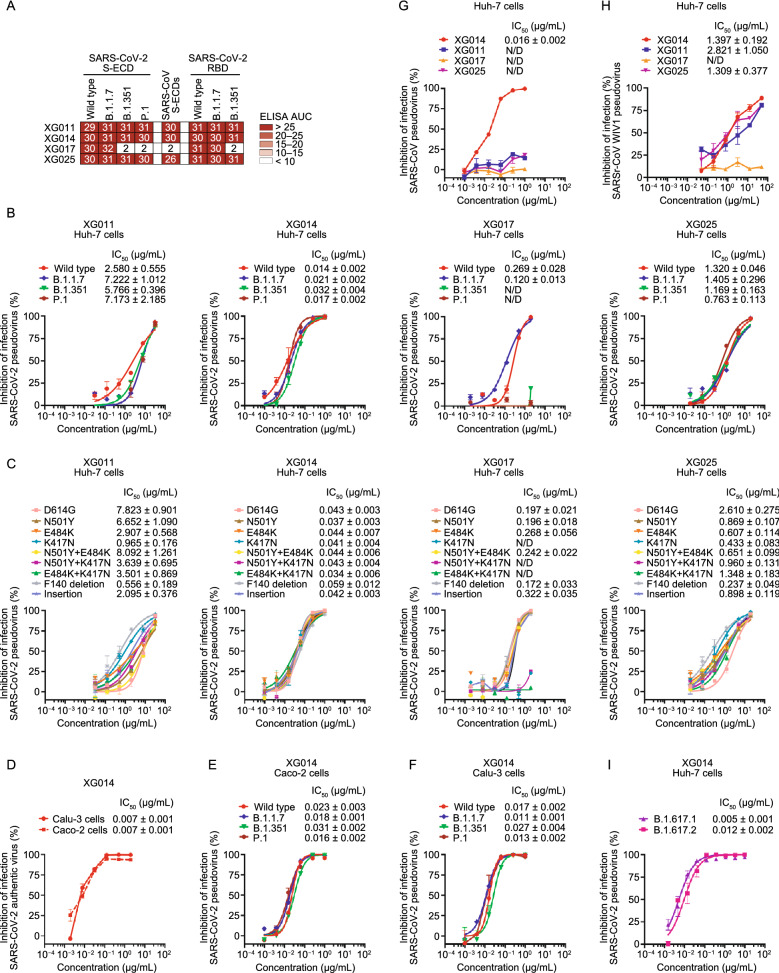
***In vitro* neutralization by monoclonal antibodies**. (A) Antibody binding to recombinant S-ECD or RBD proteins or SARS-CoV-2, its variants and SARS-CoV. The area under the curve (AUC) values were calculated by PRISM software (AUC). Experiments were performed at least twice. (B and C) *In vitro* neutralization assays using SARS-CoV-2 pseudoviruses in Huh-7 cells. Percent inhibition of infection in the presence of the indicated antibodies XG011, XG014, XG017, and XG025 is shown normalized to infection without antibody addition. Insertion: an 11-amino-acid insertion between Y248 and L249 (KTRNKSTSRRE). Data are shown as mean ± SEM. N/D, not detected. (D–F) *In vitro* neutralization assays using SARS-CoV-2 authentic virus (D) or pseudoviruses (E and F) in Caco-2 and Calu-3 cells. Percent inhibition of infection by different concentrations of XG014 are shown as means ± SEM. (G and H) *In vitro* neutralization assays using SARS-CoV (G) or SARSr-CoV WIV1 (H) pseudoviruses in Huh-7 cells. Percent inhibition of infection is normalized to infection without antibody addition. Data are shown as mean ± SEM. (I) *In vitro* neutralization assays using SARS-CoV-2 pseudoviruses (B.1.617.1 and B.1.617.2 strains) in Huh-7 cells. Percent inhibition of infection in the presence of the antibodies XG014 is shown normalized to infection without antibody addition. Data are shown as mean ± SEM. All *in vitro* neutralization experiments in this figure were performed at least two times

To assess whether these four monoclonal antibodies recognize newly emerging SARS-CoV-2 variants, we performed enzyme-linked immunosorbent assays (ELISAs) using the recombinant S-ECD (extracellular domain of S protein) or RBD proteins of SARS-CoV-2 and its related variants. XG011, XG014 and XG025 maintained binding comparable to wildtype when tested against the S-ECD of B.1.1.7, B.1.351, and P.1 strains and the RBD of B.1.1.7 and B.1.351 (Fig. 2A). Antibody XG017, on the other hand, despite normal binding capacity against the RBD and S-ECD of B.1.1.7, failed to bind to the S-ECD of B.1.351 and P.1 and the RBD of B.1.351, likely attributed to the mutations contained in the RBD region (Fig. 2A).

To investigate whether these four antibodies could still neutralize various SARS-CoV-2 variants, we developed several pseudoviruses expressing mutants of SARS-CoV-2 S protein (Fig. S1A). Neutralization assays using similar levels of SARS-CoV-2 pseudoviruses and its mutants (Fig. S1A) revealed that the IC_50_ values ranged from 2.580–7.222 µg/mL for XG011, 0.014–0.032 µg/mL for XG014, and 0.763–1.405 µg/mL for XG025 (Fig. 2B). XG014 exhibited broad and potent neutralizing activity against all three variants, while XG017 failed to neutralize SARS-CoV-2 pseudovirus variants B.1.351 and P.1 in line with the ELISA binding data (Fig. 2B). We further tested the neutralization activity against pseudoviruses bearing single or double mutations (see MATERIALS AND METHODS). Results showed that XG011, XG014, and XG025 effectively neutralized these pseudoviruses with IC_50_ values ranging from 0.556–8.092 µg/mL, 0.034-0.059 µg/mL and 0.237–2.610 µg/mL, respectively (Fig. 2C). In contrast, XG017 exhibited no neutralizing activity against pseudoviruses containing a K417N mutation (Fig. 2C). Together, the epitopes of XG011, XG014 and XG025, but not XG017, appear to be conserved among tested SARS-CoV-2 circulating variants.

Lungs and intestines are the main organs for SARS-CoV-2 infection (Lamers et al., 2020). To examine the neutralizing activity of XG014 in the context of different cell types, we selected a human lung cell line, Calu-3, and a cell line originating from human intestine, Caco-2, to perform *in vitro* neutralization assays using both authentic viruses and pseudoviruses. Consistent with the authentic virus experiments in Vero-E6 cells (Fig. 1C), XG014 potently inhibited authentic SARS-CoV-2 infection in Calu-3 and Caco-2 cells with IC_50_ values of 0.007 µg/mL in both cell setups (Fig. 2D). We were also able to visualize complete or partial suppression of SARS-CoV-2 N protein expression mediated by XG014 through an immunofluorescence assay (Fig. S1B and S1C). Moreover, we also assessed XG014 against SARS-CoV-2 variant pseudoviruses in these two cell line systems, which again showed no reduction of XG014 neutralization in both Calu-3 and Caco-2 cells (Figs. 2E, 2F, S1D and S1E).

To investigate epitope conservation across different β-CoVs, the neutralizing activities of these four monoclonal antibodies against SARS-CoV and SARS-related coronavirus (SARSr-CoV) WIV1 pseudoviruses were measured. Only XG014, but not XG011, XG017 or XG025, neutralized SARS-CoV pseudovirus with IC_50_ values of 0.016 µg/mL in Huh-7 cells or 0.024 µg/mL in ACE2-overexpressing A549 (A549-ACE2) cells, respectively (Figs. 2G and S1F). XG014 also inhibited the infection by bat SARSr-CoV WIV1 pseudovirus in Huh-7 and ACE2-overexpressing A549 cells (Figs. 2H and S1G). Collectively, combining with the results that XG014 efficiently neutralized the newly emerging SARS-CoV-2 variants B.1.617.1 and B.1.617.2 (Fig. 2I), these results suggested that XG014 exhibits exceptionally potent and broad neutralizing activity against β-CoV-B, including SARS-CoV-2, its variants, SARS-CoV, and bat SARSr-CoV WIV1 *in vitro*.

### Structural analysis of SARS-CoV-2 S-XG014 complex

To characterize the epitope of XG014 precisely and understand its cross-neutralization mechanism, we solved the structure of the prefusion stabilized SARS-CoV-2 S ectodomain trimer complexed with XG014 using single-particle cryo-EM. The complex structure was refined to an overall 3.4 Å resolution with the RBD-XG014 interface region locally refined using a “block-based” reconstruction approach to 3.9 Å resolution (Fig. S2).

**Figure 3:**
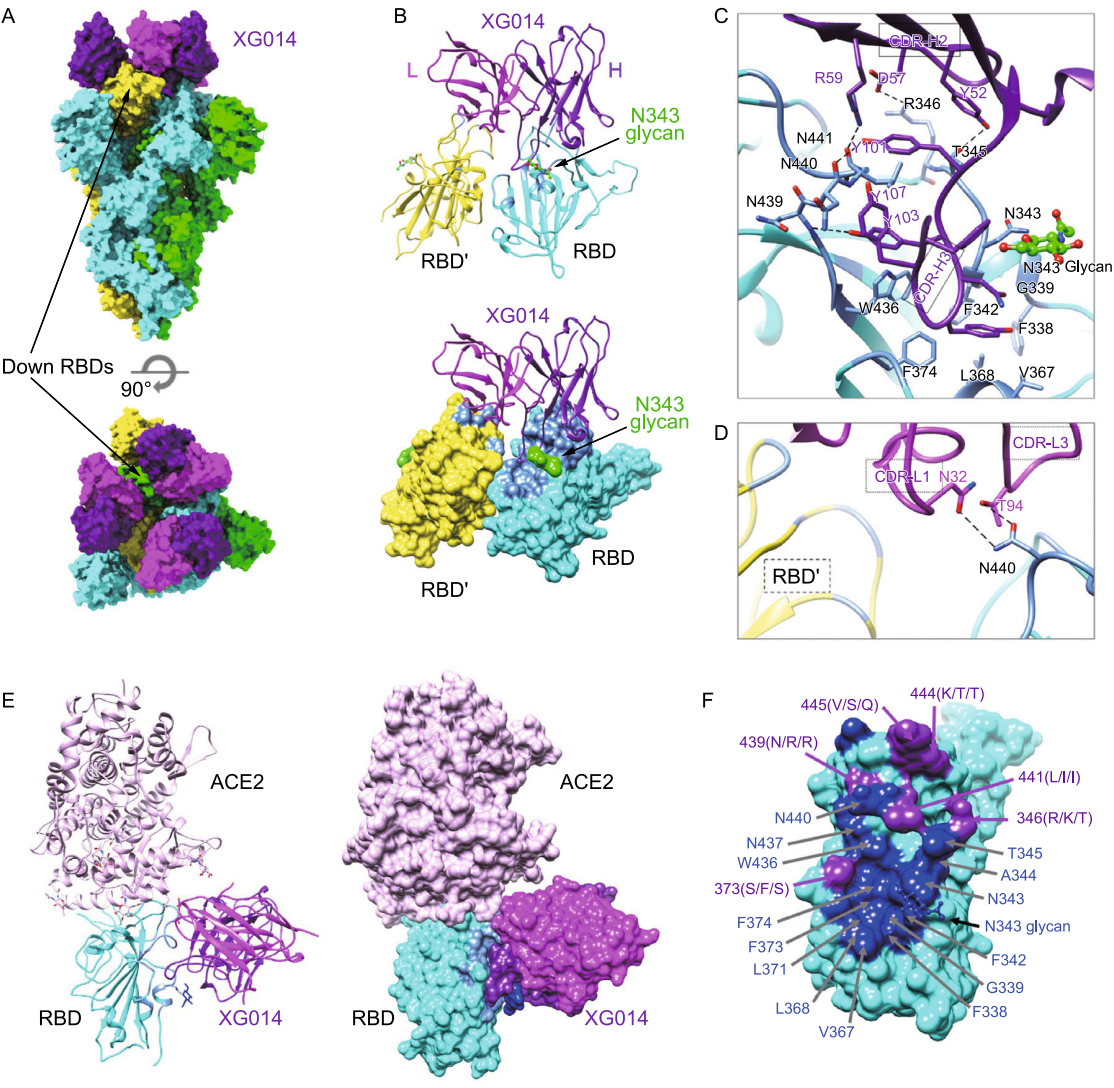
**Cryo-EM structure of the SARS-CoV-2 S trimer complexed with XG014 fab**. (A) Molecular surface representation of the SARS-CoV-2 S-XG014 complex structure with three RBDs “down”, viewed along two orthogonal orientations. Each SARS-CoV-2 protomer is colored distinctly (cyan, green and yellow). Exemplary RBDs in “down” conformation are indicated by arrows. XG014 light and heavy chains are colored magenta and purple, respectively. (B) Cartoon representation of XG014-RBD regions. (C) Key interactions between XG014 heavy chain and SARS-CoV-2 RBD. Hydrogen bonds are represented by dashed lines. (D) Key interactions between XG014 light chain and SARS-CoV-2 RBD. (E) Positioning of ACE2 (pink) relative to the XG014 bound to SARS-CoV-2 RBD. (F) Molecular surface representation of the SARS CoV-2 RBD (cyan). Residues involved in the XG014 interactions are labeled and colored according to its conservation with SARS-CoV and bat SARSr-CoV WIV1. The conserved and variable residues are colored blue and purple, respectively

The complex structure revealed that three XG014 Fabs bound to a completely closed S trimer with all RBDs at “down” conformation (Fig. 3A). Since the ligand-free form of the S trimer used in this study mostly shows one “up” RBD and two “down” RBDs (Wrapp et al., 2020), this result suggested that XG014 stabilized or induced a closed S-trimer conformation. Further analysis showed that XG014 binds to the apex of the closed S trimer and targets the core RBD region. The binding of XG014 to RBD buried ~908 Å^2^ of surface area, with the heavy chain and light chain contributing ~701 and 207 Å^2^, respectively (Fig. 3B). The interaction between the heavy chain and RBD is primarily due to extensive hydrophobic interactions. The 16-residue-long hydrophobic complementarity-determining region CDR-H3 (HQYGYNYGYFYYYIDV) inserts into a hydrophobic cavity formed by the RBD residues F338, F342, V367, L368, F374, W436, L441 and the glycan linked to N343 residue (Fig. 3C). In addition, hydrophilic interactions further enhance XG014 binding by forming hydrogen bonds between CDR-H2 and both T345 and R346, as well as CDR-H3 and residues 439–441. Besides, the residues N32 on CDR-L1 and T94 on CRD-L3 of the light chain are also involved in the interaction by forming two hydrogen bonds with N440 (Fig. 3D), which is strictly conserved among SARS-CoV-2, SARS-CoV and SARSr-CoV WIV1 (Fig. S3).

A notable feature of XG014 is that it also contacts the adjacent RBD by its light chain and buries an additional 62 Å^2^ of surface area (Fig. 3B). This contact is mainly by Van der Waals interactions, without specific hydrogen bonds or hydrophobic interactions. Moreover, structural modeling suggested that XG014’s major epitope does not overlap with the ACE2 binding sites (Fig. 3E).

The structural data also aligns well with the cross-neutralizing activity of XG014 against β-CoV-B, including SARS-CoV-2 variants, SARS-CoV and bat SARSr-CoV WIV1, as most key contact residues are conserved (Figs. 3F and S3). Residues 417, 484 and 501, identified as key mutation sites in recently circulating SARS-CoV-2 variants (Grubaugh et al., 2021; Supasa et al., 2021; Xie et al., 2021), are not involved in the interactions with XG014. Consequently, XG014 retains its neutralizing activity towards these recent SARS-CoV-2 variants. Among 20 residues involved in the major interactions between XG014 and RBD, 14 residues are strictly conserved, and one residue (L144I) is similar (Figs. 3F and S3). Among the variable residues, N439 (R in SARS-CoV and SARSr-CoV WIV1) interacts with XG014 only by its main chain, so the interaction is not affected by residue substitution. A notable variable residue is R346, which forms a salt bridge with XG014 (Fig. 3C). In SARS-CoV, the salt bridge remains when this Arginine residue is replaced by a lysine, which is a similar basic residue (Fig. S3). However, in SARSr-CoV WIV1, the arginine is substituted by a threonine, which will abolish the salt bridge and might partially affect the XG014 Fab binding (Fig. S3). This is consistent with the observation that a higher concentration of XG014 is required to neutralize SARSr-CoV WIV1 than that required to neutralize SARS-CoV.

### XG014 has a unique antigen-binding epitope

**Figure 4:**
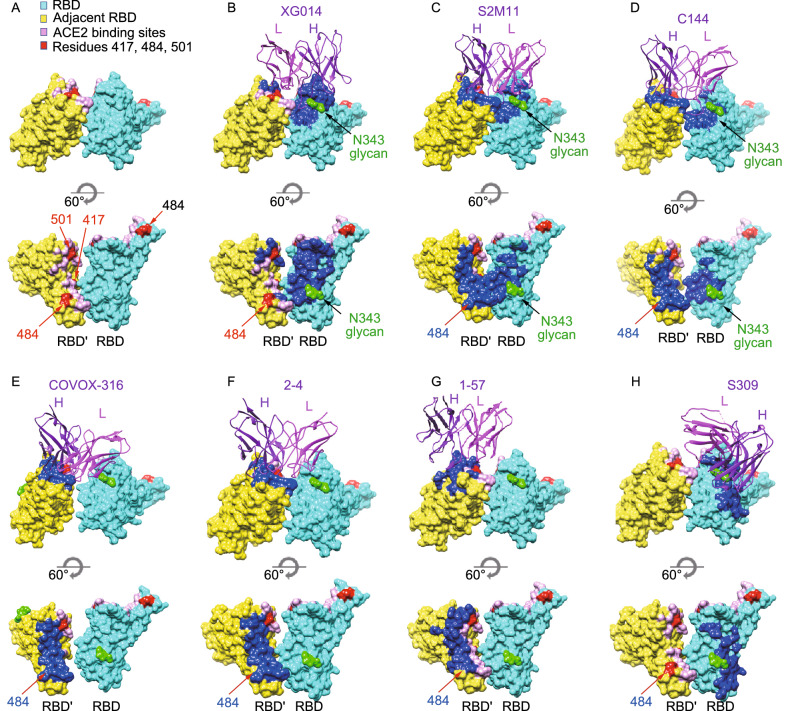
**Comparison of XG014 epitopes with other antibodies that bind all-“down” SARS-CoV-2 S trimers**. (A) Surface representation of two adjacent RBDs (labeled as RBD and RBD’) in all closed S trimers. ACE2 binding residues are colored pink, and the hot mutation sites 417, 484 and 501 are colored red. (B–H) Surface representation of two adjacent RBDs, in all closed S trimers, including XG014 (this study) (Zhou et al., 2021b), S2M11 (PDB ID: 7K43) (Tortorici MA, 2020), C144 (PDB ID: 7K90) (Barnes et al., 2020), COVOX-316 (PDB ID: 7ND7) (Dejnirattisai et al., 2021), 2-4 (PDB ID: 6XEY) (Liu et al., 2020a), 1-57 (PDB ID: 7LS9) (Cerutti et al., 2021) and S309 (PDB ID: 6WPS) (Pinto et al., 2020). The epitopes for each indicated antibody are colored as blue

Given the unusual all-“down”-RBD conformation of S trimers induced by XG014 (Fig. 4A and 4B), we asked what features of XG014’s binding mode might explain its preference for closed S trimers. First, XG014 makes contacts with the N343 glycan and it has been proposed that the N343 glycan acts as a “glycan gate” to control the opening of the SARS-CoV-2 S protein (Sztain et al., 2021). Therefore, XG014’s binding to N343 glycan might be involved in blocking the opening of RBD and thus completely locking S trimer at the closed state. The second notable feature of XG014 is its quaternary interactions with two adjacent RBDs. Structural modeling suggested that XG014’s major epitope is accessible in both the open and closed RBD states and does not overlap with the ACE2 binding sites (Fig. 3E). Based on the modeling, XG014 should be able to bind to “up” RBDs as long as it does not engage the neighboring RBD through its light chain. It therefore seems that the XG014 light chain attachment to the neighboring RBDs is a possible mechanism underlying the arrangement of all RBDs to the “down” or closed state. In summary, it could be concluded that XG014 mediates neutralization by locking the SARS-CoV-2 S trimer in a closed state to block the attachment of the receptor, which only recognizes open RBDs.

To better understand the mechanism behind S trimer closure, we investigated more antibodies/nanobodies that have been shown to only bind to all-“down” S trimers, including S2M11 (Tortorici MA, 2020) (Fig. 4C), C144 (Barnes et al., 2020) (Fig. 4D), COVOX-316 (Dejnirattisai et al., 2021) (Fig. 4E), 2-4 (Liu et al., 2020a) (Fig. 4F) and 1-57 (Cerutti et al., 2021) (Fig. 4G). Remarkably, all five of these antibodies target RBM epitopes in contrast to XG014 (Fig. 4B–G; Table 1). S2M11 nanobody and C144 Fab span two neighboring RBDs and thereby rely on quaternary interactions (Fig. 4C and 4D). Other than the RBM epitopes on one RBD, both S2M11 and C144 insert their CRD-H3s into the hydrophobic patch of the neighboring RBD. This patch is formed by residues 338–342, 367–368 and 436, which happens to overlap with XG014 epitopes. Notably, the N343 glycan is also involved in S2M11 and C144 binding, which might also partially explain their closed S trimer interaction. Antibodies COVOX-316, 2-4 and 1-57, on the other hand, only target one RBD and do not make contacts with neighboring monomers (Fig. 4E and 4F), suggesting that the quaternary interaction is not necessarily required for antibodies to preferentially interact with closed S trimers. In this comparison, we also included S309, a well-studied SARS-CoV-2 antibody, which binds to both all-“down” S trimers and one-“up”-RBD S trimer (Pinto et al., 2020). S309 targets a region that partially overlaps with XG014 epitopes, including N343 glycan (Fig. 4H). Interestingly, it is observed that S309 epitope is away from the interface of the two adjacent RBDs, while the epitopes of all the other all-“down” antibodies are close to the interface or span two RBDs (Fig. 4H). This implies that an epitope close to the next RBD or spanning two RBDs might induce S trimer closure and stabilize it in a close state. Overall, both targeting of the N343 glycan and quaternary interactions across multiple RBDs are likely relevant for S trimer closure. It seems plausible that the antibodies spanning two neighboring RBDs would stabilize the closed S trimers more efficiently.

**Table 1:** **Comparison of antibodies that bind all-“Down” SARS-CoV-2 S trimers**. N/D, not determined

	IC_50_	Cross-neutralization activities		Overlapping with RBM region	Overlapping with escape mutations such as E484	Block all RBD in the "down" conformation	Interaction with 1 or 2 RBDs simultaneously
**S2M11** (Tortorici MA, 2020)	SARS-CoV-2 3 ng/mL	SARS-CoV	-	Yes	Yes	**Yes**	**Two**
		SARSr-CoV WIV1	N/D				
		B.1.351	-				
		P.1	-				
**C144** (Barnes et al., 2020)	SARS-CoV-2 2.55 ng/mL	SARS-CoV	-	Yes	Yes	**Yes**	**Two**
		SARSr-CoV WIV1	-				
		B.1.351	-				
		P.1	-				
**COVOX-316** (Dejnirattisai et al., 2021)	SARS-CoV-2 10 ng/mL	SARS-CoV	N/D	Yes	Yes	**Yes**	One
		SARSr-CoV WIV1	N/D				
		B.1.351	-				
		P.1	-				
**2-4** (Liu et al., 2020a)	SARS-CoV-2 57 ng/mL	SARS-CoV	N/D	Yes	Yes	**Yes**	One
		SARSr-CoV WIV1	N/D				
		B.1.351	-				
		P.1	-				
**1-57** (Cerutti et al., 2021)	SARS-CoV-2 8 ng/mL	SARS-CoV	N/D	Yes	Weak interaction	**Yes**	One
		SARSr-CoV WIV1	N/D				
		B.1.351	+				
		P.1	+				
**S309** (Pinto et al., 2020)	SARS-CoV-2 79 ng/mL	SARS-CoV	+	**No**	**No**	No	One
		SARSr-CoV WIV1	+				
		B.1.351	+				
		P.1	N/D				
**XG014** (Zhou et al., 2021b)	SARS-CoV-2 7 ng/mL	SARS-CoV	+	**No**	**No**	**Yes**	**Two**
		SARSr-CoV WIV1	+				
		B.1.351	+				
		P.1	+				

The major epitope residues recognized by XG014 are outside the hotspots of RBM, where the prevalent mutations, such as K417N, E484K, N501Y substitutions, are located (Fig. 4B). On the contrary, the antigenic surface of antibodies S2M11, C144, COVOX-316, 2-4, and 1-57 overlaps with E484 residue (Fig. 4C–G), making them fail to neutralize B.1.351 and P.1 SARS-CoV-2 variants, which include E484K mutation (Table 1). Taken together, the quaternary epitope of XG014 appears to be unique compared to other RBM-specific neutralizing antibodies, making XG014 an attractive candidate for therapeutic use.

### Antibodies targeting the same epitope group as XG014

**Figure 5:**
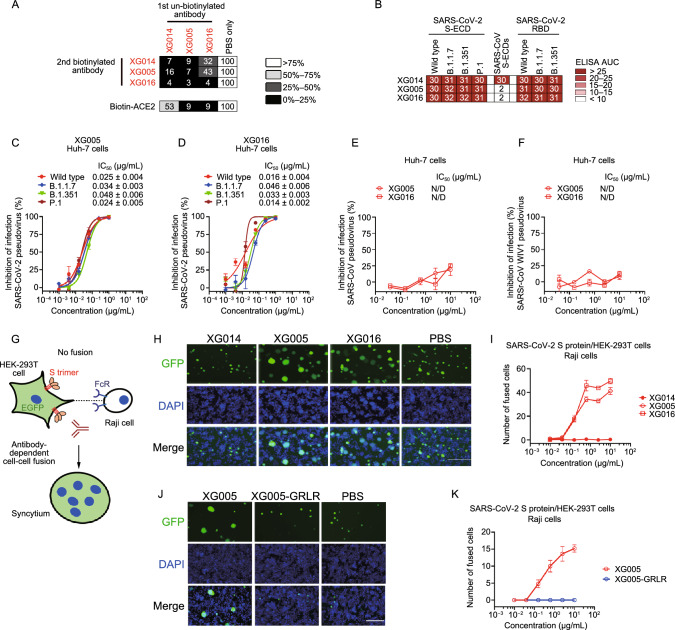
**XG005 and XG016, but not XG014, induce S protein-mediated membrane fusion**. (A) Competition ELISA for three neutralizing antibodies (XG014, XG005 and XG016) targeting the same epitope group. Results of competition ELISA shown as percent of binding by biotinylated antibodies and illustrated by colors: black, 0%–25%; dark gray, 26%–50%; light gray, 51%–75%; and white, >76%. Representative of two experiments. (B) Antibody binding to recombinant S-ECD or RBD proteins or SARS-CoV-2, its variants and SARS-CoV. The area under the curve (AUC) values were calculated by PRISM software (AUC). (C and D) *In vitro* neutralization assays using SARS-CoV-2 pseudoviruses in Huh-7 cells. Percent inhibition of infection by XG005 (C) or XG016 (D) is shown normalized to infection without antibody addition. Data are shown as mean ± SEM. All experiments were repeated at least twice. (E and F) *In vitro* neutralization assays using SARS-CoV (E) or SARSr-CoV WIV1 (F) pseudoviruses in Huh-7 cells. Percent inhibition of infection is normalized to infection without antibody addition. Data are shown as mean ± SEM. N/D, not detected. (G) Schematic diagram of antibody-dependent S protein-mediated membrane fusion between Raji cells expressing Fc gamma receptor II (FcγRII) and HEK-293T cells overexpressing SARS-CoV-2 S protein. (H) XG005 and XG016, but not XG014 and PBS, promoted syncytium formation between Raji cells and HEK-293T cells overexpressing SARS-CoV-2 S protein. Scale bar for fluorescent images, 200 µm. (I) Numbers of fused cells induced by different concentrations of the indicated antibodies. (J) Fluorescent images of SARS-CoV-2 S protein-mediated membrane fusion in the presence of XG005 or XG005-GRLR. Scale bar, 200 µm. (K) Numbers of fused cells induced by XG005 and XG005-GRLR. All *in vitro* neutralization and cell-cell fusion experiments in this figure were performed at least two times

The unique epitope of XG014 led us to functionally investigate the antibodies belonging to the same epitope group. XG005 and XG016 competitively bind to RBD with XG014 (Zhou et al., 2021b), and no simultaneous binding of RBD by these three antibodies was observed (Fig. 5A).

Although XG005, XG016 and XG014 equally recognized the S-ECD and RBD proteins of SARS-CoV-2 variants, B.1.1.7, B.1.351, and P.1, only XG014 bound to the S-ECD of SARS-CoV (Fig. 5B). Consistently, XG005 and XG016 efficiently neutralized SARS-CoV-2 pseudovirus and its variants (Fig. 5C and 5D), but not SARS-CoV or SARSr-CoV WIV1 pseudoviruses (Fig. 5E and 5F), suggesting a decreased level of cross-neutralizing activity compared with XG014.

The fusion of SARS-CoV-2-infected cells with neighboring uninfected cells has been widely observed in the late stage of SARS-CoV-2 infection, and antibodies are reported to inhibit or accelerate S protein-mediated membrane fusion (Bussani et al., 2020). Our established cell-cell fusion assay (Fig. S4A) has shown a significant plasma membrane fusion induced by HEK-293T cells overexpressing SARS-CoV-2 or SARS-CoV S protein (Xia et al., 2020) (Fig. S4B). Using this assay, we showed that XG014 efficiently inhibited cell-cell fusion mediated by S protein of SARS-CoV-2 or SARS-CoV (Fig. S4B and S4C), and did so in a manner independent of host cell type (Fig. S4D and S4E).

To evaluate the potential effect of antibody-dependent S protein-mediated member fusion, we further established a cell-cell fusion assay using the ACE2-negative but SARS-CoV-2 S protein-overexpressing HEK-293T cells and the Fc gamma receptor II (Fc $\gamma$ RII)^+^/ACE2^−^ Raji cells, a human B lymphoblast cell line (Fig. 5G). Both XG005 and XG016 induced the membrane fusion between these two cell lines in a dose-dependent manner (Fig. 5H and 5I). In contrast, similar to the PBS treatment control group, XG014 had no effect on membrane fusion (Fig. 5H and 5I). Abrogating antibody-FcγR interaction through mutations within the Fc region of XG005 (XG005-GRLR; G236R and L328R mutation) (Horton et al., 2008; Zhou et al., 2021b) abolished the induction of S protein-mediated membrane fusion (Fig. 5J and 5K).

We then used EK1, a peptide targeting HR1 domain of S protein to block six-helix bundle formation and thus inhibit the SARS-CoV-2 S protein-mediated membrane fusion (Xia et al., 2020). The presence of EK1 completely blocked S protein-mediated membrane fusion in a dose-dependent manner, emphasizing the crucial role of SARS-CoV-2 S protein during the S protein-mediated membrane fusion (Fig. S4F and S4G). Together, these results indicated that some neutralizing antibodies, such as XG005 and XG016, belong to the same epitope group with XG014, but exhibit a lower level of cross-neutralizing activity than XG014 and can induce S protein-mediated membrane fusion, effects not observed for XG014.

### Structural comparison between XG005- and XG014-bound SARS-CoV-2 S trimers

Competition ELISA using recombinant ACE2 protein suggested that XG005 and XG016, but not XG014, blocked the interaction of SARS-CoV-2 RBD with the cellular receptor ACE2, suggesting an overlapping but distinct antigen-binding mode among these three antibodies (Fig. 5A).

**Figure 6:**
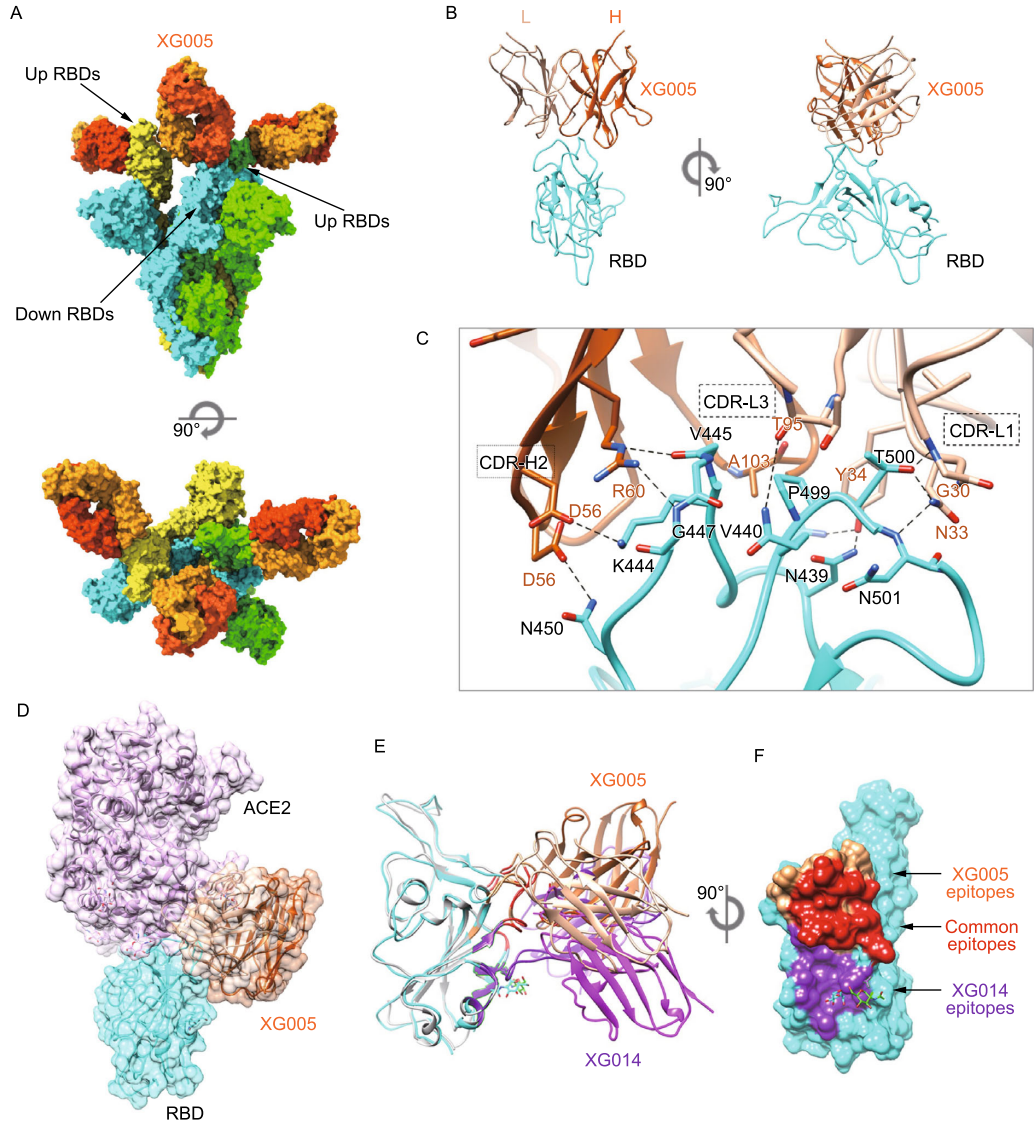
**Cryo-EM structure of SARS-CoV-2 S trimer complexed with XG005**. (A) Molecular surface representation of the SARS-CoV-2 S-XG005 complex structure with three RBDs “down”, viewed along two orthogonal orientations. Each SARS-CoV-2 protomer is colored distinctly (cyan, green and yellow). The XG005 light and heavy chains are colored light orange and dark orange, respectively. (B) Cartoon representation of XG005-RBD regions. (C) Key interactions between XG005 and SARS-CoV-2 RBD. Hydrogen bonds are represented by dashed lines. (D) Positioning of ACE2 (pink) relative to the XG005 bound to SARS-CoV-2 RBD. (E) Comparison of XG014-RBD (XG014: purple; RBD: light gray) and XG005-RBD (XG005: orange; RBD: cyan) structures. (F) Molecular surface representation of the SARS CoV-2 RBD (cyan), showing the difference and similarity of XG005 and XG014 epitopes. The common residues involved in both XG005 and XG014 interactions are colored red. The specific residues involved in XG005 or XG014 binding are colored orange or purple, respectively

To further understand their structural difference, we determined the cryo-EM structure of the SARS-CoV-2 S trimer complexed with XG005 Fab to an overall 3.8 Å resolution (Fig. 6A). To improve the resolution of these regions, each RBD/XG005 Fab interface region was locally refined individually to a resolution 4.2–4.4 Å (Fig. S5). Similar to XG014, XG005 bound all three RBDs. In contrast to the XG014 structure, however, most XG005-bound S particles exhibited two “up” RBDs and one “down” RBD conformation (Fig. 6A). The binding of the XG005 Fab molecules with the RBD domain buried ~813 Å^2^ of surface area, with the heavy chain and light chain contributing 480 and 333 Å^2^, respectively. The XG005-RBD interaction focused on two loop regions of RBD (L437-450 and L498-502), with the latter overlapping with the RBM (Fig. 6B and 6C). The interaction with RBD was mediated by CDR-L1 and CRD-L3 on the light chain and CDRH3, CDRH2 and the following β strand (59–61) of the heavy chain (Fig. 6C). In total 20 residues in the RBD are involved in the binding to XG005 (Fig. S3). The tight contacts primarily result from extensive hydrophilic interactions. Intensive hydrogen bonds and salt bridges were formed between CDR-H2, CDR-L1 on XG005 and N450, K444, V445, G447, V440, N439, T500 and N501 of RBD (Fig. 6C). Additionally, hydrophobic contacts from CDR-L3 (A102, A103) and P499 from RBD further enhance the XG005 interactions (Fig. 6C).

As predicted based on the competition assay (Fig. 5A), the RBD epitope of XG005 shares common residues with the RBD epitope of XG014, mostly located at the one-loop region (L437-450), and overlaps with the ACE2 binding site (Fig. 6D and 6E). Both XG005 and XG014 interact with this region by hydrophilic interactions, including hydrogen bonds and salt bridges. Additionally, XG014 and XG005 each have their own unique epitopes, with XG014 targeting an extra conserved hydrophobic patch distant from RBM, while XG005 directly targets less conserved ACE2 binding residues (Fig. 6E and 6F), illustrating different neutralizing mechanisms and characteristics.

The most common conformation observed for the unbound S construct we used is one “up” and two “down” RBDs (Wrapp et al., 2020). ACE2 can only recognize “up” RBDs, which are less stable and favor conversion of S to the post-fusion state. Structural modeling revealed that XG005 binding requires the opening of at least two RBDs, suggesting that XG005 induces the SARS-CoV-2 S conformational equilibrium toward opened RBDs. In contrast, XG014 induces all RBDs to the closed conformation, thereby locking S trimer at a stable prefusion state and limiting its conformational change, which is essential for receptor attachment, viral entry and membrane fusion. This helps explain why XG005 promotes S protein-mediated membrane fusion, while XG014 did not. Summarizing, even though XG005 and XG014 belong to the same epitope group, the specific epitope regions towards or away from the RBM region are responsible for their different functional properties of XG005 and XG014, even though they share partially common epitopes.

### Protection and treatment in human ACE2 transgenic mice

**Figure 7:**
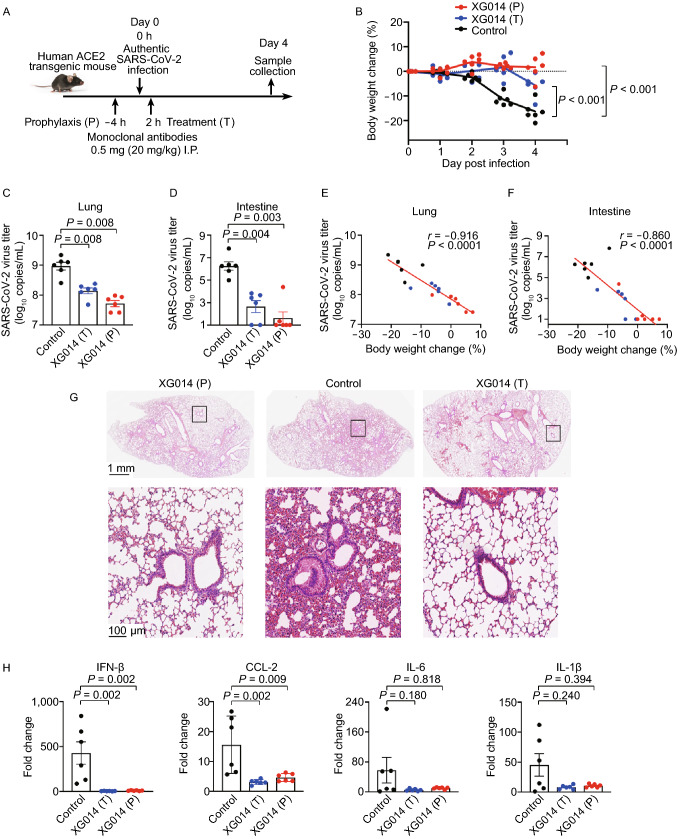
**XG014 is protective and therapeutic against SARS-CoV-2 *in vivo***. (A) Diagram of prophylaxis and treatment protocols. (B) Body weight changes in prophylaxis, treatment and control groups. (C and D) SARS-CoV-2 viral mRNA titers in lungs (C) and intestines (D) four days post-challenge. RT-qPCR was used to quantify the SARS-CoV-2 viral mRNA load. (E and F) Dot plot showing the correlations between body weight changes (x axis) and SARS-CoV-2 viral mRNA titers (y axis) in lungs (E) and intestines (F). Spearman’s rank correlation coefficient (r) and significance value (*P*). (G) Histopathological staining of mouse lungs from the prophylaxis, treatment, and control groups. (H) mRNA levels of cytokines and chemokines, such as IFN-β, CCL-2, IL-6, and IL-1β in lungs four days post-infection. The statistical differences (*P* values) in this figure were all determined by Wilcoxon rank-sum test

To evaluate whether XG014 could be developed as a preventive or therapeutic antibody against SARS-CoV-2 infection, we used hACE2-Tg mice and administered antibodies intraperitoneally prior to or after authentic SARS-CoV-2 challenge (Fig. 7A). Mild, or no, body weight loss was observed for both prophylaxis and treatment groups (Fig. 7B). In contrast, mice in the control group suffered severe body weight loss (Fig. 7B). XG014 administration significantly reduced SARS-CoV-2 viral RNA load in both lungs and intestines (Fig. 7C and 7D), the levels of which were inversely correlated with the loss of weight (*r* = −0.9156, *P* < 0.0001 for lungs; *r* = −0.8601, *P* < 0.0001 for intestines) (Fig. 7E and 7F). Typical viral interstitial pneumonia in the control group was observed in lungs, while the XG014-treated transgenic mice in both prophylaxis and treatment groups displayed only limited pathological lung changes (Fig. 7G). Consistently, mRNA levels encoding several cytokines, such as IFN-β and CCL-2, in the lungs were decreased after XG014 treatment, compared with the mice in the control group (Fig. 7H). Together, these results suggest that a single administration of XG014 could provide effective protection and therapeutic effect against SARS-CoV-2 infection *in vivo*.

## DISCUSSION

SARS-CoV-2 belongs to the class of positive-stranded RNA viruses, which are intrinsically prone to mutational change (Zhou et al., 2021a). To date, SARS-CoV-2 variants of B.1.1.7 (Alpha), B.1.351 (Beta), P.1 (Gamma) and B.1.617.2 (Delta) have spread globally and raised serious concerns over the efficacy of vaccines and monoclonal antibodies (Zhou et al., 2021a; Wang et al., 2021b). The RBD region, especially the RBM motif, is responsible for binding to host receptor ACE2, thus containing multiple conformational neutralizing epitopes for blocking ACE2 interaction (Du et al., 2009; Liu et al., 2020b; Zhou et al., 2021b). Interestingly, mutations in the RBD, especially the interaction surface between ACE2 and RBD (RBM motif), not only increase receptor affinity, thus leading to a higher transmissibility, but also escape antibody immune responses. Among these mutations, the N501Y substitution, identified in all B.1.1.7, B.1.351 and P.1 variants, enhances transmissibility (Starr et al., 2020; Tegally et al., 2021), and the E484K and K417N/K417T, found in B.1.351 or P.1 variants, are associated with the antibody escape phenotype (Greaney et al., 2021; Madhi et al., 2021; Chen et al., 2021b). The RBM is reported to be highly variable in these variants (Thomson et al., 2021), so antibodies targeting an epitope outside of RBM could offer enhanced activity against numerous escape variants.

In this study, we identified and functionally, as well as structurally, defined a highly conserved antibody epitope on S protein, which is located outside the RBM. Antibody XG014, which targets this epitope, exhibits strong resistance against escape mutations located in the RBM, thus neutralizing B.1.1.7, B.1.351, P.1 and B.1.617.2 pseudoviruses. Moreover, XG014 neutralizes SARS-CoV and bat SARSr-CoV WIV1, suggesting that XG014 targets a neutralizing epitope conserved across multiple β-CoVs. Combined with the findings that XG014 maintains binding potency against RBD mutants, including V341I, F342L, V367F, R408I, A435S, G476S and V483A (Zhou et al., 2021b), and that XG014 is protective and therapeutic, we conclude that XG014 recognizes a potent and broad cross-neutralizing epitope outside of RBM in RBD.

Our structural findings provide mechanistic insights into how XG014 neutralize SARS-CoV-2 and accommodates antigenic variation. Specifically, XG014 locks all RBDs at the “down” state and thus blocks receptor attachment and viral entry indirectly, as only “up” RBD can recognize ACE2. Most residues that constitute the epitope are conserved, while the key mutation sites 417, 484 and 501, as identified in the recently isolated SARS-CoV-2 variants, are not involved in XG014 binding. Some mutations at the epitope region, such as V341I, F342L, V367F, had no effect on Fab binding, because these mutations did not change the surface of the hydrophobic cavity, which forms major interactions with XG014’s heavy chain. Besides, residues 338–346 (FGEVFNATR) of RBD and the N343 glycan important for XG014 interaction make extensive contacts in several antibodies (316, S2M11 and Fab 309), making 338–346 an epitope hotspot.

Recently, one nanobody has been reported to simulate ACE2-RBD binding, to trigger conformational changes and to activate the SARS-CoV-2 fusion machinery (Koenig et al., 2021). We thus speculated that some antibodies, like XG005 and XG016, might drag SARS-CoV-2 virions to the host cells via their interaction with Fc receptor and conformationally change S protein to induce virion-cell fusion. This is supported by our observation that most XG005-bound SARS-CoV-2 S trimer particles have two “up” RBDs, even though the most common conformation for the S construct we used in our study has only one “up” RBD and two “down” RBDs (Wrapp et al., 2020). Thus, XG005 induces a rearrangement of RBDs to the “up” state, which is essential for receptor attachment and further conformational changes required for membrane fusion and viral entry. In contrast, XG014 induced all RBDs to the “down” state, thereby limiting the conformational change of RBD. These distinct characteristics explain their differential abilities to promote the virion-cell fusion.

Cell-cell fusion is mediated by the viral S protein in the S protein-expressing cells (or SARS-CoV-2-infected cells) and ACE2 receptor on ACE2-expressing cells together, but could also be independent of ACE2 receptor and through the Fc $\gamma$ R-mediated pathway. Similar to some antibodies against dengue virus (DENV), respiratory syncytial virus (RSV), human immunodeficiency virus (HIV) and other viruses (Arvin et al., 2020; Wen et al., 2020; Su et al., 2021), antibodies against SARS-CoV-2 S protein, such as XG005 and XG016, also seem to induce antibody-dependent viral entry (Wu et al., 2020; Zhou et al., 2021b) and S protein-mediated membrane fusion in Fc $\gamma$ RII-bearing Raji cells. However, XG014 belonging to the same epitope group does not induce overt ADE effect due to its distinct mechanism of action.

Conformational plasticity of immunogens plays an important role in vaccine design. For example, the introduction of two consecutive proline residues could stabilize the S protein of β-CoV in the prefusion conformation and thus significantly improved their immunogenicity (Pallesen et al., 2017). This strategy has been widely applied in the development of the COVID-19 vaccines (Dagotto et al., 2020). From the antigenic surface targeted by the XG014, we speculate that vaccination with the stabilized S protein with all RBD in the “down” state might induce more antibodies like XG014, thus increasing the breadth and potency of the induced neutralizing antibodies. Notably, S309, another cross-neutralizing antibody against SARS-CoV-2 and SARS-CoV, also locks RBD in the “down” conformation, further illustrating that multiple neutralizing epitopes exist that are associated with a locking of the RBD in the “down” state. Since it has been reported that a disulfide linkage formed by a double cysteine mutant (S383C and D985C) could lock all the RBD in the “down” state (Henderson et al., 2020), to use such RBD-locked-down S proteins as a vaccine to boost the immune response could be a promising strategy for broader cross-neutralizing antibody responses.

In summary, the conserved epitope outside of RBM targeted by XG014 implies that this antigenic surface could be developed as a promising target for the rational design of safe pan-vaccines against β-CoV-B. Moreover, the ultrapotent broadly neutralizing monoclonal antibody XG014 represents a promising candidate for the prevention or treatment of infections by SARS-CoV-2, SARS-CoV-2 variants, SARS-CoV and potentially new SARSr-CoVs.

## Materials and methods

### Cell lines

HEK-293T cells, Vero-E6 cells, Huh-7 cells, Calu-3 cells and Caco-2 cells were obtained from the American Type Culture Collection (ATCC), and A549 cells stably expressing ACE2 (A549/ACE2) were obtained from Dr. Xuanming Yang at the School of Life Sciences and Biotechnology, Shanghai Jiao Tong University. These cells were propagated in Dulbecco’s Modified Eagle’s Medium (DMEM) (Invitrogen, USA) containing 10% fetal bovine serum (FBS) (Gibco, USA). Raji cells (ATCC) were maintained in RPMI-1640 supplemented with 10% FBS.

### Antibodies expression

All cloned human monoclonal antibodies and their GRLR version were expressed by transient transfection to HEK-293F cells as previously reported. Briefly, HEK-293F cells were maintained in OPM 293 CD05 culture medium (OPM Biosciences, China) and transiently co-transfected plasmids encoding heavy chain or light chain using EZ Trans transfection reagents (Life iLAB Bio-Technology, China). After seven days, suspensions were harvested, and antibodies were purified using Protein G resin. Purified antibodies were electrophoresed by using SDS-PAGE. All antibodies were stored at 4 °C.

### BLI

BLI was carried out on an Octet RED96 system (ForteBio) to analyze binding kinetics of monoclonals against SARS-CoV-2 RBD (Kactus Biosystems, China). The measurements were performed using streptavidin (SA) biosensors. Antigen as well as monoclonal antibodies were diluted with PBST (PBS, 0.02% TritonX-100) in a black 96-well plate (Greiner Bio-One). For immobilizing antigens onto the SA biosensor surface, Avi-tagged SARS-CoV-2 RBD was biotinylated using the BirA biotinylation kit (Avidity). Prior to each assay, SA biosensors were pre-wetted in distilled water for at least 10 min. All steps were performed at 30 °C with shaking at 1,000 rpm. Five steps were carried out for each experiment, including (1) equilibration in water (60 s); (2) immobilization of RBD-biotin (10 µg/mL) to the sensor (loading, 300 s); (3) baseline in PBST (120 s); (4) association of monoclonals at four concentrations from 2.47–66.7 nmol/L for measurement of K_on_ (300 s); and (5) dissociation of monoclonals for measurement of K_dis_ (300 s). Curves were analyzed using Data Analysis software (ForteBio).

### ELISA and competition ELISA

Both ELISA and competition ELISA were performed as described previously (Liu et al., 2020b; Zhou et al., 2021b). ELISA plates were coated with 10 µg/mL of S-ECD or RBD of wild-type SARS-CoV-2 (Kactus Biosystems, China), or its variants, including B.1.1.7, B.1.351 and P.1 in phosphate-buffered saline (PBS) overnight at 4 °C. Plates were then blocked with 2% bovine serum albumin (BSA) in PBS. Antibodies were three-fold serially diluted with a maximum concentration of 10 µg/mL for eight dilutions and incubated for one hour at room temperature. Detection was performed after incubating with HRP-conjugated goat anti-human IgG for one hour at room temperature. The area under the curve (AUC) was calculated by using PRISM software. For competition ELISA, plates were coated with 2 µg/mL of SARS-CoV-2 RBD (Kactus Biosystems, China) and incubated with 15 µg/mL of 1st unbiotinylated antibodies for two h at room temperature. 2nd biotinylated antibodies (0.25 µg/mL) were directly added and incubated for 30 min at room temperature. Detection was performed using streptavidin-HRP (BD Biosciences). Signals generated by the 2nd biotinylated antibodies, but without 1st antibody blocking (PBS buffer substituted for the 1st blocking antibody), were used as a reference for normalization.

### Generation of pseudotyped viruses

SARS-CoV-2, SARS-CoV-2 variants, SARS-CoV, and bat SARSr-CoV WIV1 pseudotyped viruses were generated as described previously (Xia et al., 2020; Liu et al., 2020b). Briefly, HEK-293T cells were transfected with the backbone plasmid of pNL4-3.Luc.R-E- and the plasmid of pcDNA3.1-SARS-CoV-2-S, or pcDNA3.1-SARS-CoV-S or pcDNA3.1-SARSr-CoV-WIV1-S using the transfection reagent, VigoFect (Vigorous Biotechnology, China). Fresh DMEM containing 10% FBS was used to replace the cell supernatants at 8 h post-transfection. The cell supernatants containing pseudoviruses were collected 60-h post-transfection, aliquoted and stored at −80 °C. Plasmids pcDNA3.1-SARS-CoV-2-S for SARS-CoV-2 variants were generated using a site mutation kit (Yeasen, China). The pseudotyped SARS-CoV-2 B.1.1.7 variant used in this study contains mutations, including 69H, 70V, and 144Y deletions, and N501Y, A570D, P681H, T716I, S982A, and D1118H substitutions. The SARS-CoV-2 B.1.351 variant used in this study contains mutations, including K417N, E484K, N501Y substitutions. The pseudotyped SARS-CoV-2 P.1 variant used in this study contains mutations, including L18F, T20N, P26S, D138Y, R190S, K417T, E484K, N501Y, H655Y, T1027I, and V1176F substitutions. The pseudotyped SARS-CoV-2 B.1.617.1 variant used in this study contains mutations D111D, E154K, L452R, E484Q, D614G, P681R, Q1071H and H1101D. The pseudotyped SARS-CoV-2 B.1.617.2 variant used in this study contains mutations T19R, G142D, EFR156-158G, L452R, T478K, D614G, P681R and D950N. Western blot against S protein was performed to quantify the pseudotyped SARS-CoV-2 and SARS-CoV-2 mutants. Rabbit anti-S protein antibody (1:3,000) (Sino Biological, China) was used as primary antibody before incubating with the HRP-coupled donkey anti-rabbit antibody (1:3,000) (Dako, Denmark). A chemiluminescent substrate (Meilun, China) was used to visualize immunoreactive bands.

### *In vitro* neutralization assay using pseudotyped viruses

*In vitro* neutralization assays using pseudoviruses were performed as described previously (Liu et al., 2020b; Zhou et al., 2021b). Briefly, Huh-7, Caco-2, and Calu-3 cells were used for infection by the pseudotyped SARS-CoV-2 or its variants, and seeded in a 96-well plate. Antibodies XG011 (maximum concentration, 32 µg/mL), XG014 (maximum concentration, 1 µg/mL), XG017 (maximum concentration, 2 µg/mL) and XG025 (maximum concentration, 20 µg/mL) were serially diluted 1:4 in DMEM medium for six dilutions in total, followed by incubation with pseudovirus soup for 30 min and then added into the Huh-7, Caco-2 or Calu-3 cells. After 12 h, we replaced the cell supernatants with fresh DMEM containing 2% FBS and cultured for another 48 h. Finally, the cells were lysed and subjected to luciferase activity measurement using a Firefly Luciferase Assay Kit (Promega, USA) and a microplate reader (Infinite M200PRO, Switzerland). For SARS-CoV and SARSr-CoV WIV1 pseudoviruses, neutralization assays were performed using Huh-7 cells or A549-ACE2 cells as described above.

### S protein-mediated cell-cell fusion in Huh-7, Caco-2 and Calu-3 cells

Cell-cell fusion assays mediated by SARS-CoV-2 or SARS-CoV S protein were conducted as described previously (Xia et al., 2020; Liu et al., 2020b). Briefly, either pAAV-IRES-EGFP-SARS-CoV-2-S or pAAV-IRES-EGFP-SARS-CoV-S plasmid was transfected into HEK-293T cells using the transfection reagent VigoFect. The HEK-293T cells overexpressing S protein of SARS-CoV-2 or SARS-CoV were incubated with different concentrations (1:4 serially diluted) of XG014 (maximum concentration, 30 µg/mL) for 30 min, followed by adding into the seeded Huh-7 or Caco-2 or Calu-3 cells. Specially, the SARS-CoV S-mediated cell-cell fusion occurs only in the presence of trypsin (80 ng/mL), while it is not required for SARS-CoV-2 S-mediated cell-cell fusion. Cells were treated with 4% paraformaldehyde after 5-hour incubation, and the numbers of fused cells were counted in five randomly selected fields using a fluorescence microscope (Nikon Eclipse Ti-S).

### Antibody-dependent S protein-mediated cell-cell fusion in Raji cells

Raji cells were used to determine the enhanced cell-cell membrane fusion by antibodies (XG005, XG014, XG016 or XG005-GRLR). Raji cells were seeded into the 0.01% poly-L-lysine-treated 96-well plates. Serially diluted (1:4) antibodies (maximum concentration, 10 µg/mL) were mixed with HEK-293T cells overexpressing SARS-CoV-2 S protein and incubated for 30 min at 37 °C. The mixture was applied onto the Raji cells and cultured for further 24 h. Cells were fixed and subjected for counting the number of fused cells in five randomly selected fields under a fluorescence microscope. EK1 Inhibition of the antibody-mediated cell-cell fusion was conducted as described previously (Wu et al., 2020). EK1 peptide (SLDQI NVTFL DLEYE MKKLE EAIKK LEESY IDLKE L) (Xia et al., 2020), synthesized at Kangbei Bio Co., Ltd. (Ningbo, China), was dissolved in water and stored at −20 °C until use. Briefly, 5 µg XG005 with different concentrations of EK1 were incubated with the S-overexpressing HEK-293T cells for 30 min before adding onto Raji cells.

### *In vitro* neutralization assay using authentic SARS-CoV-2 virus

Authentic SARS-CoV-2 (GenBank: MT121215.1) was provided by the Shanghai Medical College, Fudan University. The viruses were amplified and titered in Vero-E6 cells using the plaque assay. Experiments including viral infections were conducted in a Biosafety Level 3 (BSL-3) laboratory of Fudan University. An *in vitro* neutralization assay was performed as described previously (Liu et al., 2020b; Zhou et al., 2021b). Briefly, Caco-2 and Calu-3 cells were seeded into a 96-well plate. Different concentrations of XG014 were mixed with the authentic SARS-CoV-2 for 30 min before adding onto the Caco-2 and Calu-3 cells. Forty-eight hours later, the supernatants were collected for quantification of the SARS-CoV-2 mRNA viral titer. Meanwhile, the cells were fixed with 4% paraformaldehyde for immunofluorescence. Reverse-transcription quantitative PCR (RT-qPCR) was used to test the SARS-CoV-2 mRNA viral titer by using the One-Step PrimeScrip RT-PCR Kit (Takara, Japan). For immunofluorescence, the rabbit anti-N antibody (Sino Biological, China) and the Alexa Fluor 488-conjugated donkey anti-rabbit IgG (Thermo Fisher Scientific) were used for observation by a fluorescence microscope (Nikon Eclipse Ti-S).

### Plaque reduction assay

Vero-E6 cells were seeded into a 96-well plate. After 24 h, XG011 (80 µg/mL), XG014 (0.16 µg/mL), XG017 (3.2 µg/mL) or XG025 (16 µg/mL) antibodies were serially diluted 1:4 in DMEM medium for six dilutions in total and incubated with authentic SARS-CoV-2 viruses for 30 min. The mixture was subsequently applied to the Vero-E6 cells and further incubated for 2 h. Subsequently, 1% carboxymethyl cellulose (Sigma, USA) was added followed by culture for further 72 h. Finally, PBS containing 4% paraformaldehyde and 1% crystal violet was added for fixation and staining. After rinsing with water, plaques were counted and the percent of plaque reduction was normalized using a PBS-treated sample.

### Expression and purification of SARS-CoV-2 S trimer

For the production of SARS-CoV-2 S ectodomains, the gene encoding the ectodomain (residues 1–1,208, GenBank: MN908947) of SARS-CoV-2 S protein with mutations at the furin cleavage site (“GSAS” substitution at residues 682–285) and S2 domain (“PP” substitution at residues 986–987), and additions of the T4 fibritin trimerization motif and a twin-strep-II tag at the C terminus was synthesized and inserted into the mammalian expression vector pCAGGS for expression as described previously (Wrapp et al., 2020). The plasmid was transiently transfected into HEK293F cells using polyethyleneimine. The supernatant was harvested after 72 h and purified by affinity chromatography. The S protein was further purified by using Superose 6 Increase 10/300 column (GE Healthcare) in 20 mmol/L Tris pH 8.0, and 200 mmol/L NaCl.

### Cryo-EM sample preparation and data collection

For cryo-grids preparation, the purified S protein and XG014 Fab were mixed at a 1:1.5 molar ratio and incubated on ice for 10 seconds to obtain the cryo-EM sample with an overall concentration of 0.7 mg/mL. Then, 3.0 μL aliquot of the sample was placed onto the freshly glow-discharged 300-mesh holey carbon-coated gold grid (C-flat, 1.2/1.3, Protochips Inc.), which was blotted by filter paper for 7 seconds and plunged into liquid ethane using Vitrobot (FEI). Afterward, the grids were transferred to liquid nitrogen for preservation. A dataset was collected with a FEI Titan Krios at 300 kV equipped with a Gatan K2 Summit direct detector. Movies (32 frames, each 0.2 s, total dose of 60 e^−^/Å^2^) were recorded with a defocus range from −1.5 to −2.7 μm, and data were automatically acquired using SerialEM (Mastronarde, 2005) with a pixel size of 1.04 Å.

To obtain SARS-CoV-2 S-XG005 complex, the S trimer was mixed with XG005 Fab at a 1:1.7 molar ratio (S monomer: Fab), incubated at 4 °C for 1 h and further purified by Superose 6 Increase 10/300 column (GE Healthcare). The peak tube was concentrated to 0.4 mg/mL in 20 mmol/L Tris pH 8.0, and 200 mmol/L NaCl. Cryo-EM data were collected on a Titan Krios microscope (Thermo Fisher) operated at 300 kV, equipped with a K2 summit direct detector (Gatan) and a GIF quantum energy filter (Gatan) setting to a slit width of 20 eV. Automated data acquisition was carried out with SerialEM software (Mastronarde, 2005). Movies (36 frames, each 0.2 s, total dose of 53 e^−^/Å^2^) were taken in the super-resolution mode at a nominal magnification 130,000×, corresponding to a physical pixel size of 1.046 Å, and a defocus range from −1.2 to −2.2 μm.

### Data processing of SARS-CoV-2 S-XG014 complex

A total of 2,977 micrographs were recorded and subjected to beam-induced motion correction in the Relion 3.0 package (Zivanov et al., 2018). The defocus values of the images were calculated by Gctf (Zhang, 2016). Then, using the map of S protein complexed with H014 (EMD-30326) as model, 577,143 particles were picked and extracted for reference-free 2D alignment. Based on this, 414,922 particles were selected and used for 3D classification without imposing symmetry, resulting in four distinct conformations with one taking account of ~84%, which was further processed by auto-refinement with the symmetry of C3 and postprocessing to generate the model of S-XG014 complex. To improve the resolution of S-XG014 binding interface, we used the block-based reconstruction strategy. The regions of the S-XG014 particles comprising RBD and XG014 were blocked and a local reconstruction of the binding interface was performed to obtain the final resolution of the focused interface. The resolution of each structure was determined on the basis of the gold-standard Fourier shell correlation (threshold = 0.143) and evaluated by ResMap (Kucukelbir et al., 2014). All procedures were performed using Relion 3.0 and details of the dataset processing were summarized in Fig. S2.

### Data processing of SARS-CoV-2 S-XG005 complex

All the data processing was carried out using either modules on, or through, RELION v3.0 (Zivanov et al., 2018). A total of 1,858 movie stacks were binned 2 × 2, dose weighted, and motion corrected using MotionCor2 (Zheng et al., 2017). Parameters of contrast transfer function (CTF) were estimated by using Gctf (Zhang, 2016). All micrographs then were manually selected for further particle picking upon ice condition, defocus range and estimated resolution.

Particles were initially auto-picked by using the Laplacian-of-Gaussian method and then subjected into two-dimensional (2D) classification. The top-class averages were used as the 2D reference for template-picking, yielding 286,984 particles. Binned 2 × 2 particles (2.088 Å/pixel) were extracted and subjected to a routine process of 2D classification, 3D initial model, 3D classification and 3D auto-refinement, followed by subsequent re-centering and re-extraction binned 1 × 1 (1.046 Å/pixel). Finally, 109,304 particles were grouped and subjected to auto-refinement, CTF refinement and Bayesian polishing, yielding a density map at 3.8-Å overall resolution.

To improve resolution at three interfaces of RBD/XG005, volumes were erased in Chimera (Pettersen et al., 2004), and the regions corresponding to the NTD/RBD domains with antibody VH-VL domains (shortened to NRAb) were used to generate respective local mask (4-pixel extension, 8-pixel soft cosine edge). Three copies of particles targeted on different NRAb regions (NRAb1, NRAb2, NRAb3) were subjected to focused 3D classification without alignment by using the local mask separately. 70,995 of NRAb1 particles were selected and then subtracted for focused refinement. After carrying out post-processing with the mask of whole NRAb1 density, we got a 4.4-Å overall resolution density map. We also did post-processing with the mask of only RBD domain and antibody VH-VL variable domains, getting a 4.2-Å overall resolution density map of binding interface. In total, 32,281 of NRAb2 particles were selected and then subtracted for focused refinement, yielding a 4.5-Å overall resolution density map. 23,948 of NRAb3 particles were selected and then subtracted for focused refinement, yielding a 4.7-Å overall resolution density map. The focused refined maps of NRAbs were fitted into the XG005 trimer map and merged with it using the “vop maximum” command in UCSF Chimera (Pettersen et al., 2004).

The reported resolutions above are based on the gold-standard Fourier shell correlation (FSC) 0.143 criterion. All the visualization and evaluation of 3D density maps were performed with UCSF Chimera (Pettersen et al., 2004), and the local resolution variations were calculated using RELION (Zivanov et al., 2018). The above procedures of data processing are summarized in Fig. S5. These composite maps were used for subsequent model building and analysis.

### Model building and structure refinement

For model building of SARS-CoV-2 S trimer-XG014 complex, the structures of the apo SARS-CoV-2 S trimer (PDB ID: 6VSB), the heavy chain of Fab 38-3-11A (PDB ID: 6Z3P) and the light chain of Fab NA-45 (PDB ID: 6PZE) were manually fitted into the final map using Chimera (Pettersen et al., 2004) and further corrected manually by real-space refinement in COOT (Emsley et al., 2010). The atomic model was further refined by positional and B-factor refinement in real space using Phenix (Afonine et al., 2018).

For model building of SARS-CoV-2 S trimer-XG005 complex, SARS-CoV-2 S protein with 2 “up” RBDs was extracted from the atomic model (PDB ID: 7A29). The initial model of XG005 Fab was generated using the SWISS-MODEL website (Waterhouse et al., 2018) based on the homologous structures of VH and VL (PDB ID: 5K8A and 6KTR). The S and antibody initial models were fitted into the composite maps of SARS-CoV-2 S trimer-XG005 complex using Chimera (Pettersen et al., 2004) and then manually adjusted with COOT (Emsley et al., 2010). Several iterative rounds of real-space refinement were further carried out in PHENIX (Afonine et al., 2018).

The final models were evaluated by Molprobity (Chen et al., 2010). Details of the datasets and refinement statistics are summarized in Table S1.

### Human ACE2 transgenic mice and *in vivo* studies

Specific-pathogen-free (SPF) female human ACE2 transgenic (hACE2-Tg) mice were purchased from Shanghai Model Organisms Center (Shanghai, China), and the experiments related to animals were conducted according to institutional regulations (approval number 20190221-070; approval date 21 February 2019). Eighteen SPF hACE2-Tg mice were randomly assigned to three groups with six mice in each group, defined as the PBS control, prophylaxis and treatment groups. A single administration of 20 mg/kg (approximately 0.5mg) XG014 was administered intraperitoneally 4 h before and 2 h after SARS-CoV-2 challenge for the prophylaxis and treatment experiments, respectively. An equal volume of PBS was administered for the control group. All mice were infected intranasally with 0.5 × 10^5^ PFU SARS-CoV-2 authentic viruses. Mouse body weight was monitored for five consecutive days. Lungs and intestines were collected 4 days post-infection. RT-qPCR was used to quantify SARS-CoV-2 viral mRNA levels. The isolated RNAs were reverse-transcribed using a first-strand cDNA synthesis kit (Takara, Japan) and amplified by real-time PCR using the SYBR Green PCR kit (Takara, Japan). The gene of glyceraldehyde 3-phosphate dehydrogenase (GAPDH) was used as an internal control for measuring the levels of cytokines. Collected lungs fixed in 4% paraformaldehyde were subjected to hematoxylin and eosin (H&E) staining.

### Statistical analysis

The detailed information of statistical analysis could be found in the Results and Figure Legends. Correlation was evaluated by Spearman’s rank correlation method (Fig. 7E and 7F). Statistical significance was calculated by Wilcoxon rank sum test (Fig. 7B–D and 7H). The ELISA area under the curve (AUC) values (Figs. 2A and 5B) were calculated in PRISM software. The IC_50_ values by neutralization assays (Figs. 1C, 2B–H, 5C and 5D) were calculated by nonlinear regression analysis in PRISM software.

## Supplementary Information

Below is the link to the electronic supplementary material.

Supplementary file1 (PDF 1130 kb)
