# Supplementary material for: An ultrapotent pan-β-coronavirus lineage B (β-CoV-B) neutralizing antibody locks the receptor-binding domain in closed conformation by targeting its conserved epitope
Source: Protein Cell. 2021 Sep 23;13(9):655–75. doi: 10.1007/s13238-021-00871-6 (PMC8458794; doi:10.1007/s13238-021-00871-6)
Supplement: Supplementary file 1 — Supplementary file1 (PDF 1130 kb) [file 13238_2021_871_MOESM1_ESM.pdf]

SUPPLEMENTARY FIGURES AND LEGENDS

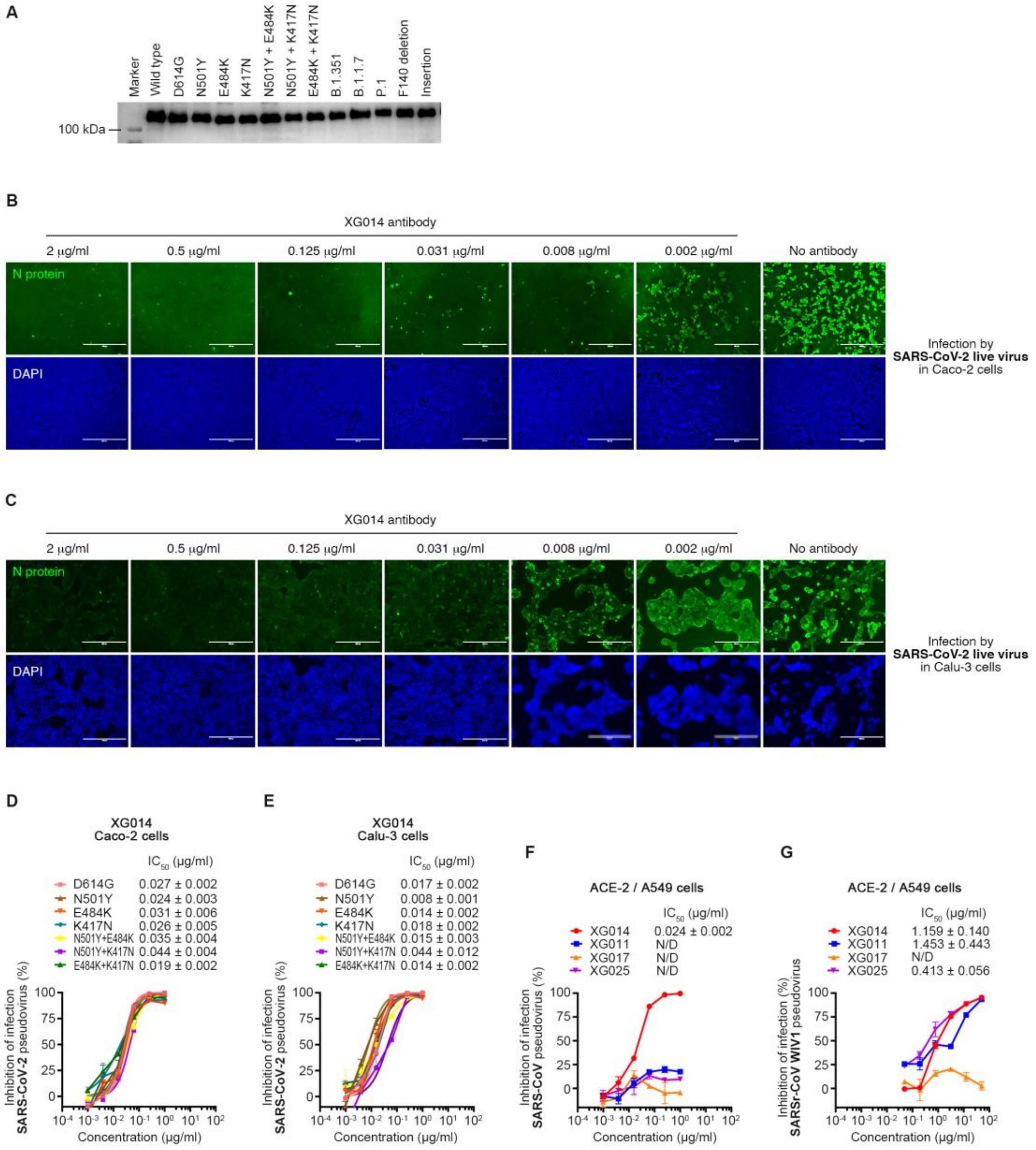

Figure S1. ***In Vitro*** **Neutralization Assays.**

(A) Quantification of the spike (S) protein for the SARS-CoV-2 variants pseudoviruses. To accurately evaluate the neutralization activity of monoclonal antibodies, Western blot analysis was adopted to quantify the SARS-CoV-2 S protein to guarantee a similar amount used for pseudoviruses.

(B-C) Immunofluorescence staining of SARS-CoV-2 N protein for *in vitro*

neutralization assays against authentic SARS-CoV-2 virus. Immunofluorescence using anti-N protein polyclonal antibodies (Zhou et al., 2021b) was used to evaluate the neutralizing effect of XG014. Scale bar, 400 μm.

(D and E) *In vitro* neutralization activity of XG014 against pseudotyped SARS-CoV-2 variants in Caco-2 (D) and Calu-3 cells (E). Experiments were performed at least twice and duplicates of neutralization are presented as mean ± SEM.

(F and G) *In vitro* neutralization assays using SARS-CoV (F) or SARSr-CoV WIV1 (G) pseudoviruses in ACE2-overexpressing A549 cells. Percent inhibition of infection is normalized to infection without antibody addition. Data are shown as mean ± SEM. All experiments were performed at least two times. N/D, not detected.

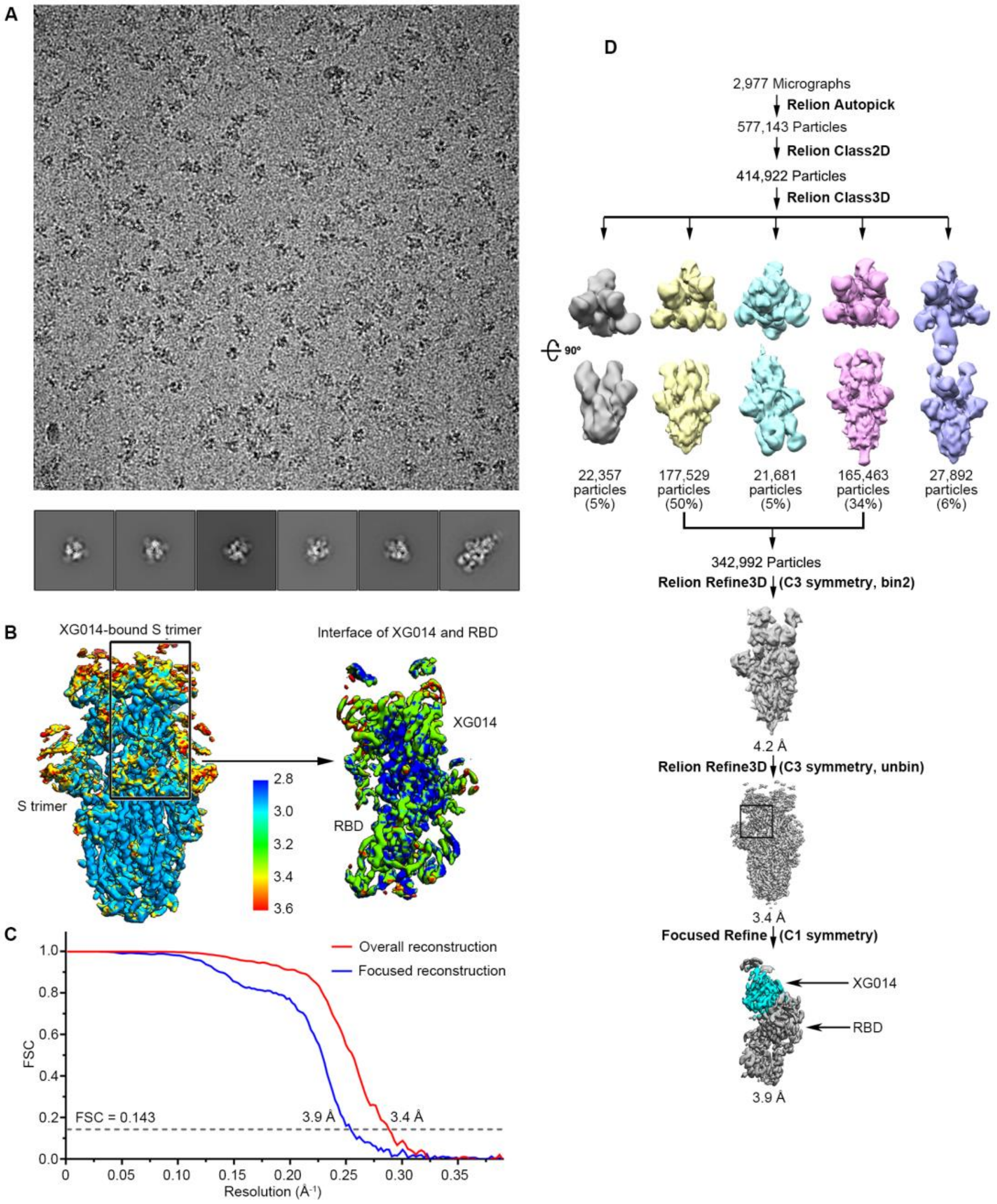

Figure S2. Cryo-EM Data Collection and Processing of XG014 Bound to SARS-CoV-2 S Trimers.

(A) Representative electron micrograph and 2D classification results of XG014 bound SARS-CoV-2 S.

(B) Local resolution map for the whole reconstruction and the locally refined RBD-014.

(C) Gold-standard Fourier shell correlation curves for the XG014-bound SARS-CoV-2

S trimer (red line) and locally refined RBD/XG014 interface region (blue line). The 0.143 cutoff is indicated by a horizontal dashed line.

(D) Data processing flowchart of XG014-bound SARS-CoV-2 S trimer.

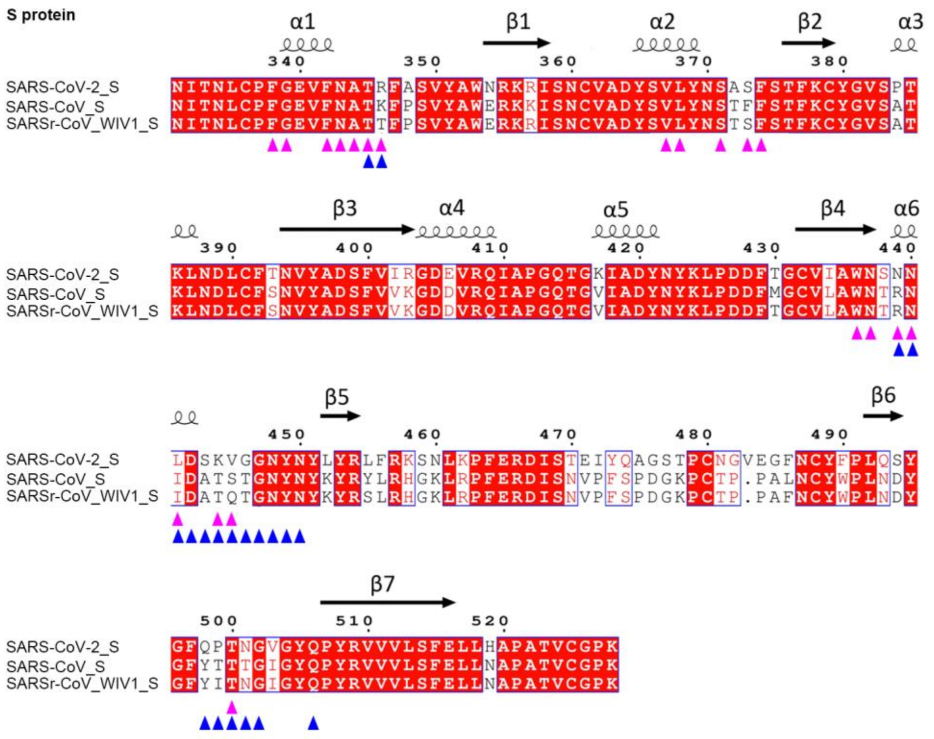

Figure S3. Amino Acid Sequence Alignment of RBD Regions.

Amino acid sequences of RBD regions from SARS-CoV-2, SARS-CoV and SARSr-CoV WIV1 were aligned. The strictly conserved amino acid residues are labeled in red. Purple and blue triangles point out the amino acid residues on the RBD involved in the major interactions for XG014 and XG005, respectively.

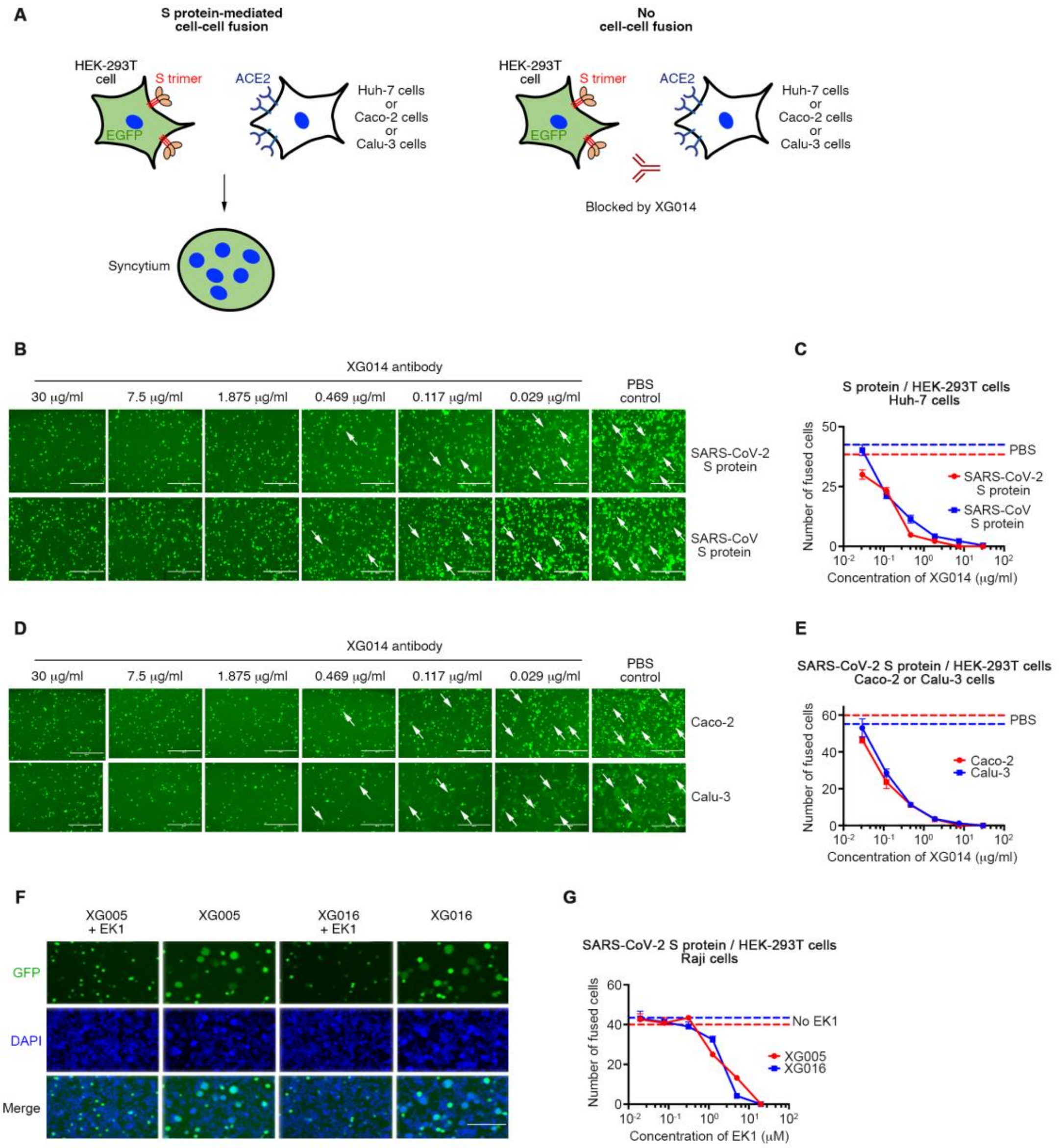

Figure S4. XG014, but not XG005 or XG016, Inhibits S Protein-Mediated Membrane Fusion.

(A) Schematic diagram of S protein-mediated membrane fusion. Blocking the S protein-ACE2 interaction by a monoclonal antibody could suppress the S protein-mediated membrane fusion between HEK-293T cells overexpressing S proteins of SARS-CoV-2 or SARS-CoV and ACE2-positive Huh-7 or Caco-2 or Calu-3 cells.

(B-C) XG014 inhibits syncytium formation between Huh-7 cells and HEK-293T cells

overexpressing S proteins of SARS-CoV-2 or SARS-CoV. Representative fluorescent images (B), scale bar, 400 μm, and fused cell numbers in the presence of XG014 (C).

(D-E) XG014 inhibits SARS-CoV-2 S protein-mediated syncytium formation in Caco-2 or Calu-3 cells. Scale bar for fluorescent images (D), 400 μm. Numbers of fused cells induced by different concentrations of XG014 (E). White arrows in (B) and (D) indicate examples of syncytia.

(F-G) Syncytium formation with Raji cells (Fig. 5G) induced by XG005 and XG016 could be efficiently inhibited by EK1. Representative fluorescent images (F), scale bar, 200 μm. Numbers of fused cells in the presence of antibodies and EK1 (G).

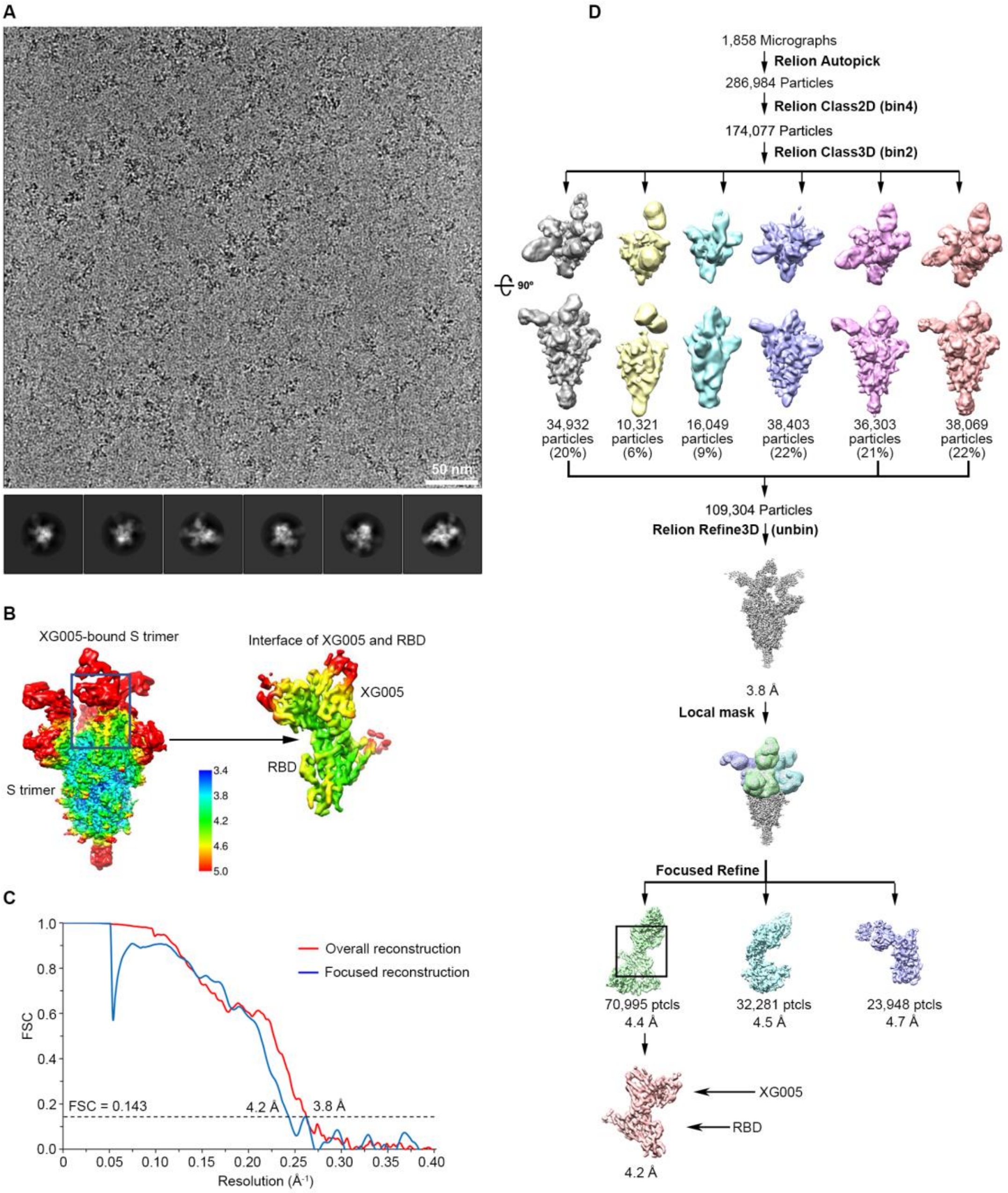

Figure S5. Cryo-EM Data Collection and Processing of XG005 Bound to SARS-CoV-2 S Trimers.

(A) Representative electron micrograph and 2D classification results of XG005-bound SARS-CoV-2 S.

(B) Local resolution map for the whole reconstruction and the locally refined RBD-XG005.

(C) Gold-standard Fourier shell correlation curves for the XG005-bound SARS-CoV-2 S trimer (red line) and locally refined RBD-XG005 interface region (blue line). The 0.143 cutoff is indicated by a horizontal dashed line.

(D) Data processing flowchart of XG005-bound SARS-CoV-2 S trimer.

Table S1. Cryo-EM Data Collection and Refinement Statistics.

Data collection and processing				
Protein	S-XG014	XG014-RBD interface	S-XG005	XG005-RBD interface
Voltage (kV)	300		300	
Detector	K2		K2	
Pixel size (Å)	1.04		1.046	
Electron dose (e^-/Å^2)	50		53	
Defocus range (μm)	1.2-2.5		1.2-2.5	
Final particles	342,992	1,028,976	109,304	70,995
Final resolution (Å)	3.4	3.9	3.8	4.2
Model refinement and validation statistics				
Ramachandran statistics				
Favored (%)	91.71		94.35	
Allowed (%)	8.12		5.35	
Outliers (%)	0.17		0.02	
Rotamer outliers (%)	0.20		0.13	
R.m.s.d.				
Bond lengths (Å)	0.006		0.002	
Bond angles (°)	0.65		0.50	
